# High Light Intensity Applied Shortly Before Harvest Improves Lettuce Nutritional Quality and Extends the Shelf Life

**DOI:** 10.3389/fpls.2021.615355

**Published:** 2021-01-28

**Authors:** Qianxixi Min, Leo F. M. Marcelis, Celine C. S. Nicole, Ernst J. Woltering

**Affiliations:** ^1^Horticulture and Product Physiology Group, Wageningen University and Research, Wageningen, Netherlands; ^2^Signify Research Laboratories, Eindhoven, Netherlands; ^3^Food and Biobased Research, Wageningen University and Research, Wageningen, Netherlands

**Keywords:** LED, lettuce, vertical farm, carbohydrates, ascorbic acid, overall visual quality, shelf life, End of Production lighting

## Abstract

The effect of light intensity applied shortly before harvest on the nutritional quality, postharvest performance, and shelf life of loose-leaf lettuce (*Lactuca sativa* L. cv. Expertise RZ Salanova^®^) was investigated. Lettuce was grown either in a greenhouse with supplemental high-pressure sodium light (Experiment 1, EXP 1) or in a climate room under white LED light (Experiment 2, EXP 2). In both experiments full grown plants were transferred to a climate room for the End of Production (EoP) light treatments during the last week of cultivation. During EoP lighting plants were exposed to different intensities (0, 110, and 270 μmol m^–2^ s^–1^ in EXP 1; 50, 210, and 470 μmol m^–2^ s^–1^ in EXP 2) from white-red LEDs for 6 (EXP 2) or 7 days (EXP 1). Mature leaves were then harvested and stored in darkness at 10°C to study the postharvest performance. Changes in dry matter content, total ascorbic acid, and carbohydrates (including glucose, fructose sucrose, and starch) levels were determined during EoP lighting and during the subsequent shelf life as indicators of lettuce nutritional quality. Quality aspects (appearance, texture, and odor) were accessed during the shelf life as indicators of postharvest performance. In both experiments, high light intensities applied in EoP lighting increased dry matter percentage and contents of ascorbic acid (AsA) and carbohydrates at harvest and these increased levels were maintained during the shelf life. Increased light intensity in EoP treatment also extended the shelf life. The levels of AsA and carbohydrates at harvest correlated positively with the subsequent shelf life, indicating that the prolonged shelf life relies on the improved energy and antioxidant status of the crop at harvest.

## Introduction

Leafy vegetables generally have a short postharvest life due to mechanical damage and the lack of light during storage and transportation. Postharvest performance is related to both nutritional quality (measured as the levels of health and flavor related compounds) and sensorial quality (accessed as visual quality scores, texture, and odor). A negative nutritional image and unattractive visual quality aspects decreases the shelf life and reduces consumer purchases of fresh products. Therefore, improving nutritional and visual quality is important for achieving a good postharvest performance.

Important nutritional elements and quality markers include the levels of carbohydrates (sucrose, fructose, glucose, and starch) and vitamin C. Carbohydrates may relate to the sensorial quality of leafy vegetables by providing sweeter or less bitter taste and delaying crop texture deterioration (shape and crispness) and discoloration (Lin et al., 2013; Hasperué et al., 2015). Vitamin C is defined as the total ascorbic acid (TAsA), which is the sum of ascorbic acid (AsA), and dehydroascorbic acid (DHA). TAsA is the major antioxidants group in leafy lettuce and is involved in balancing redox status and eliminating enzymatic pinking and browning. Hence, high levels of carbohydrates and TAsA are potentially beneficial for postharvest performance.

Both carbohydrates and TAsA levels are affected by light conditions (Ntagkas et al., 2018). The production of carbohydrates is directly related to the photosynthetic rate, which is dependent on light. Carbohydrates content at harvest significantly increases by increasing light levels during growth of leafy vegetables in both greenhouses and vertical farms (Zhou et al., 2009; Pérez-López et al., 2015). However, excess light during growth may also induce an unbalanced redox status (Zhou et al., 2009). In such a situation, TAsA is rapidly produced and acts as a strong antioxidant to scavenge the reactive oxygen species (ROS; Yabuta et al., 2007; Zhou et al., 2009, Zhou et al., 2012). Therefore, carbohydrates and TAsA levels can be increased by increasing light intensity during growth, which can potentially lead to a better postharvest performance of leafy vegetables.

Light exposure during the postharvest phase may also preserve plants nutritional quality and protects plants from visual quality deterioration (Büchert et al., 2011; Woltering and Witkowska, 2016). Postharvest lighting delayed sugars decrease, enlarged antioxidants capacity (accumulated AsA), delayed chlorophyll degradation, lowered the browning index and suppression of browning-related enzyme activities (polyphenol oxidase, PPO and peroxidase, POD) in leaves (Toledo et al., 2003; Zhan et al., 2012a, b, 2013; Hasperué et al., 2016b). However, postharvest lighting also stimulates stomata opening and may lead to loss of fresh weight and texture (Noichinda et al., 2007; Olarte et al., 2009; Hasperué et al., 2016a). If postharvest lighting has positive effects on quality of lettuce, it remains a question whether high light levels applied as End of Production (EoP) lighting can also promote postharvest quality. Lettuce harvested with improved initial quality may better resist unfavorable postharvest conditions and thus present a better postharvest performance.

The objective of this study was to investigate the effect of light treatments applied before harvest on postharvest performance in lettuce, as a representative crop of leafy vegetables. We tested two hypotheses: (1) short-term light treatment with high light intensity applied to the plants before harvest increases the carbohydrates and TAsA levels at harvest, and (2) the increased carbohydrates and TAsA levels at harvest improve postharvest visual and nutritional quality and extends the shelf life. To this end, full grown lettuce plants that were grown either in a greenhouse (EXP 1) or vertical farm (EXP 2) were subjected for up to 1 week to different EoP LED light intensities in a climate room. Lettuce leaves were sampled pre-harvest and during postharvest storage to measure the nutritional and sensorial quality traits (sucrose, fructose, glucose, starch, TAsA, and sonsorial arttrubutes) and to assess their shelf life (days till unacceptable quality).

## Materials and Methods

### Plant Material and Cultivation Conditions

Lettuce Expertise (*Lactuca sativa* L. cv. Expertise RZ Salanova^®^) was grown either in a greenhouse (EXP 1, Wageningen, Netherland) or in a climate chamber (EXP 2, Eindhoven, Netherland) for approximately 5 weeks. In both experiments, approximately 1 week before commercial harvest, plants were transferred into a climate chamber for the EoP light treatment. The main cultivation conditions for both experiments are summarized in Table 1.

**TABLE 1:** Cultivation conditions for Expertise lettuce cv. Expertise in 2 experiments.

Conditions	Experiment 1 (EXP 1)	Experiment 2 (EXP 2)
Cultivation environment	Greenhouse	Vertical farm (Climate chamber)
Substrate	Rockwool 8 cm × 8 cm × 6.5 cm	Rockwool 8 cm × 8 cm × 6.5 cm
Light intensity ^a^ in growth phase (PPFD ^b^, before EoP treatments)	237 ± 19.6 μmol m^–2^ s^–1^	210 ± 6 μmol m^–2^ s^–1^
Photoperiod	16 h	18 h
Light source in growth phase (before EoP treatments)	Natural light HPS lamps	White LED light Far-red LED light (first 4 weeks)
Density	25 plant m^–2^	32 plant m^–2^
Day/night temp	20/18°C	23/22°C
CO_2_ concentration	Ambient level	1,000 ppm
Relative air humidity (light / dark period)	–	70%/80%

*^a^Average light intensity in light period before short-term pre-harvest light treatment applied. ^b^PPFD, photosynthetic photon flux density (400–700 nm) measured at plant level.*

In EXP 1, seeds were sown in potting soil, given a 2-days pre-cold treatment at 4°C in dark and then followed by 2 weeks gemination period (from 27th January 2016 to 10th February 2016) in the greenhouse at 16°C. Uniform seedlings were selected and transplanted into rock wool cubes (8 cm × 8 cm × 6.5 cm, Grodan Rockwool B.V., Netherlands). Plants were grown in the greenhouse for 5 weeks with a density of 25 plants m^–2^. The irrigation solution contained NO_3_^–^, 10.9; NH_4_^+^, 1.2; H_2_PO_4_^–^, 1.1; K^+^, 6.12; Ca^2+^, 2.5; Mg^2+^, 0.84; SO_4_^2–^, 0.56; Cl^–^, 1.53 mmol L^–1^; Fe^3+^, 25; B^3+^, 20; Cu^2+^, 0.5; Zn^2+^, 5; Mn^2+^, 8; and Mo^+^, 0.5 μmol L^–1^ (pH = 5.8 and EC = 1.5 mS cm^–1^). This is the composition used in commercial practice when irrigation water is taken into account. Temperature was set at 20 and 18°C for day and night, respectively. The average photosynthetic photon flux density (PPFD) in the growth phase was estimated as 237 ± 19.6 μmol m^–2^ s^–1^ during the day, a value representing the daily average light intensity of the light period during the entire growth phase. This was calculated from global radiation outside the greenhouse considering 62% transmission and 50% photosynthetically active radiation. Either shading screen or high pressure sodium (HPS) light was applied 16 h a day from 5:00 am to 21:00 pm. Six plants in a group were rotated every 2 days to assure a uniform illumination over the plants.

In EXP 2, seeds were sown in rock wool plugs (Grodan Rockwool B.V., Netherlands) and germinated under LED lighting [GreenPower LED research module DR/W (Supplementary Table 1), Philips, Netherlands] with 140 μmol m^–2^ s^–1^ and 18 h photoperiod (24:00 am to 18:00 pm) for 6 days. Seedlings were then transferred into rock wool cubes (7 cm × 7 cm × 6.5 cm, Grodan Rockwool B.V., Netherlands) and grown for 5 weeks with a density of 32 plants m^–2^ and under same LED modules with intensity of 211 ± 6 μmol m^–2^ s^–1^ and a photoperiod of 18 h. Far-red light was added only during the first 4 weeks (13.6 ± 0.31 μmol s^–1^ m^–2^, Research module far-red, Philips, Netherlands). Light was measured as the mean of 15 measurements that were equally distributed over the illuminated area. Day/night temperature was set at 23/22°C. The irrigation solution was supplied through ebb and flood system and had the following composition: NO_3_^–^, 12.91; NH_4_^+^, 0.38; H_2_PO_4_^–^, 1.53; K^+^, 8.82; Ca^2+^, 4.22; Mg^2+^, 1.15; SO_4_^2–^, 1.53; Cl^–^,1.53 mmol L^–1^; Fe^3+^, 30.67; B^3+^, 38.33; Cu^2+^, 0.77; Zn^2+^, 3.83; Mn^2+^, 3.83; and Mo^+^, 0.38 μmol L^–1^ (pH = 6 and EC = 2.3 mS cm^–1^). This is the composition used in commercial practice when considered irrigation water. Daily average relative humidity was maintained at 70 and 80% for light and dark period, CO_2_ concentration was supplied at 1,000 ppm during light period.

### End of Production Light Treatments

**FIGURE 1:**
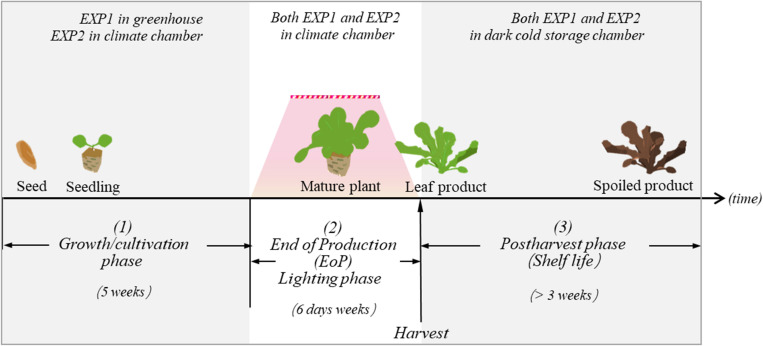
In both EXP1 and EXP2, lettuce plants were exposed to three different experimental phases: (1) the growth/cultivation phase, (2) the EoP light treatment phase, and (3) the harvest and postharvest phase. The aim of our study is to investigate how different level of light intensity in the EoP phase affects the nutritional contents and quality performance of lettuce during the postharvest phase.

**FIGURE 2:**
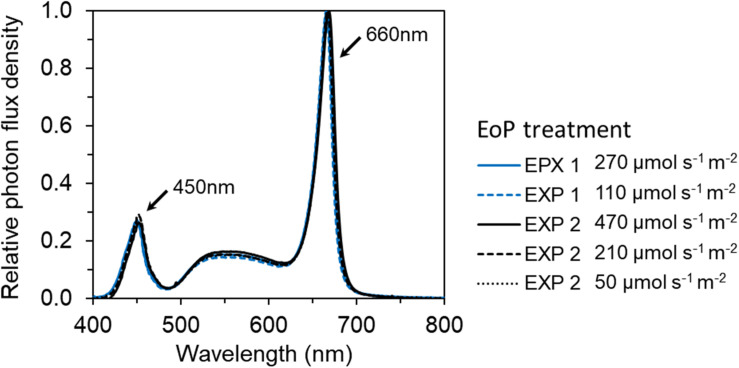
The spectral distribution of red and white LEDs (GreenPower LED toplight module DR/W_Vision MB, Philips, Netherlands) used in EoP light treatments in both EXP 1 and EXP 2. The relative photon distribution of 6 EoP light treatments were same and overlapped on each other.

In both experiments, 5 weeks old lettuce plants were transferred to the climate chamber and randomly distributed over EoP light treatments with different light intensities for 7 days in EXP 1, and 6 days in EXP 2. In both experiments, different light intensities were applied by red and white LEDs (GreenPower LED toplight module DR/W_Vision MB, Philips, Netherlands) in isolated compartments. In EXP 1 plants were held under 0 (darkness), 110 and 270 μmol m^–2^ s^–1^; in EXP 2 under 50, 210, and 470 μmol m^–2^ s^–1^ light treatments. Experimental set up and specifics of the light treatments and spectral properties are summarized in Table 2 and Figures 1, 2. Light intensity, spectrum and light distribution were measured at plant level (approximately 55 cm from LED lamps) using spectroradiometer (USB2000, Ocean Optics, Duiven, Netherlands). Light profile of EoP light treatments were measured as the mean of 25 and 15 measurements that were equally distributed over the illuminated area, respectively, in EXP 1 EXP 2. Phytochrome stationary state (PSS) was calculated by Eq. 1 (Sager et al., 1988), where *N*_λ_ stand for photon flux at wavelength λ nm, σ_*rλ*_ stands for photochemical cross-section of red absorbing phytochrome state and σ_*frλ*_ stands for photochemical cross-section of far-red absorbing phytochrome state.

(1) $$PSS=\left(\sum_{300}^{800}N_{\lambda }\sigma_{r\lambda }\right)/\left(\sum_{300}^{800}N_{\lambda }\sigma_{r\lambda }+\sum_{300}^{800}N_{\lambda }\sigma_{fr\lambda }\right)$$

**TABLE 2:** Measured light conditions during the End of Production (EoP) light treatments.

	Light source used in EoP treatments	PPFD^a^ (μ mol s^–1^ m^–2^)	PSS^b^	Photoperiod (hours)	Treatment duration (d)
EXP 1	Red and white LEDs (GreenPower LED toplight module DR/W_Vision MB, Philips, Netherlands)	0 ± 0	–	–	7
		109 ± 12.32	0.88	16	7
		266 ± 7.21	0.87	16	7
EXP 2		49 ± 1.46	0.87	18	6
		211 ± 6.02	0.87	18	6
		469 ± 17.58	0.87	18	6

*Photosynthetic photon flux density (PPFD) and phytochrome stationary state (PSS) data represent the mean of 25 measurements equally distributed over the illuminated area in each treatment compartment. ^a^PPFD, photosynthetic photon flux density (400–700 nm). ^b^PSS, phytochrome stationary state.*

Plants were rotated within each compartment every 2 days to ensure homogeneous illumination. Temperature, photoperiod, and irrigation were kept the same as the conditions during the previous growth period in each experiment. During the EoP light treatments relative humidity was 70 and 80% for light and dark period, CO_2_ concentration was at ambient level. Air temperature was measured at canopy level using *k*-type thermocouples (shielded with aluminum foil to avoid the direct radiation from LED lamps) on TC-80 data loggers (Picotechnology LETD., Cambridge, United Kingdom). The air temperature differences between each compartment were less than 1°C (data not shown).

### Leaf Sampling and Postharvest Conditions

Fully expanded lettuce leaves were sampled for carbohydrates and TAsA analysis during the EoP light treatment (day 0, 1, 4, and 7 in EXP 1 and day 0 and 6 in EXP 2) and during subsequent shelf life (day 3, 7, 10, 13, and 16 in EXP 2). Leaves were always selected from the middle “whorls” and sampled approximately 10 h after start of light period. At each sampling time, per light treatment, 16 leaves were selected randomly from 16 plants (1 leaf per plant) and pooled into 4 replicates with 4 leaves each. In each pooled sample, leaves were halved along the mid rib into two equal parts for either carbohydrates or TAsA analysis. These samples were immediately frozen in liquid nitrogen and stored at −80°C.

Additionally, four pooled samples with intact leaves were taken before (day 0) and at the end of the light treatments (day 6 or 7) to determine the dry matter content. Following weighing (fresh weight, FW), samples were oven dried at 70°C for 3 days to determine the dry weight (DW).

For overall visual quality (OVQ) and shelf life assessment, leaves harvested at the last day of the light treatment were stored in darkness at 10°C. For each light treatment, 16 mature leaves were selected randomly from 16 plants (1 leaf per plant) and pooled into 4 replicates of 4 leaves each. Each pooled sample of four leaves was assigned to one plastic box (18 L × 13 W × 6.5 H cm), where a double layered wet filter paper was placed underneath. The box was covered by a lid with 12 punched pinholes in order to maintain a high relative humidity but allow sufficient air exchange. Every day (EXP 1) or every 2 days (EXP 2), 4 sample boxes from each treatment were taken to assess the OVQ scores.

### Determination of Overall Visual Quality and Shelf Life

The OVQ of lettuce leaves was evaluated by a panel of two experienced assessors, according to ratings scale (Kader et al., 1973), modified for cultivar Expertise (Table 3). At each sampling time, sample boxes from all treatments without information on treatment were presented to assessors in a random order, at room temperature. The assessors evaluated quality parameters including appearance (yellowing, senescence browning, and wound browning/pinking), texture (crispness), and odor (smell; Supplementary Table 2). Evaluations were carried out under same white fluorescent light. All the quality parameters were scored with a structured scale from 1 (very bad) to 9 (excellent) and score 6 marking the lower limit of consumer acceptance. The shelf life was calculated as number of days from harvest till OVQ scores drop below 6.

**TABLE 3:** The description of overall visual quality (OVQ) scoring scale for lettuce leaves (cv. Expertise).

*9- Excellent*	Bright and typical natural color of leaf blade and petiole; no browning was shown; firm and crispy with fresh grass like smell. All samples meet score = 9 in appearance, texture, and odor.
*8- Very good*	One slightly discolored or browning or pinking feature are shown at the leaf cut edge or blade. Leaves are firm and crisp and fresh grass like smell. All samples meet score ≥8 in appearance, texture, and odor.
*7- Good*	Few slightly discolored leaves and brown edges are allowed. leaves still crisp, reduced fresh smell. All samples meet score ≥7 in appearance, texture, and odor.
*6- Satisfactory*	The defined consumer acceptance threshold Slightly discolored leaves and moderate brown edges are allowed. No unpleasant odor or texture decay. All samples meet score ≥ 6 and no sample below score 6 in appearance, texture, and odor.
	
*5- Mediocre*	Some yellowing and browning of leaf blade; slightly brown petiole; darker brown cut edge; texture decay but still acceptable; slightly unpleasant odor emerged. One or more samples meet score 5 in appearance, texture, and odor.
*4- Borderline*	Obvious discolouration on leaf blades; browning of leaf blade and petiole; clearly mild soft in texture; unpleasant odor. One or more samples meet score 4 in appearance, texture, and odor.
*3- Poor*	Strong discolouration, browning of leaves; wilted texture; obvious unpleasant odor. One or more samples meet score 3 in appearance, texture, and odor.
*2- Bad*	Complete yellow or brown leaf; texture decay with liquid leakage; strong off-odor. One or more samples meet score 2 in appearance, texture, and odor.
*1- Very bad*	Complete discolored leaf; liquid leaking from leaf material; fermented smell. One or more samples meet score 1 in appearance, texture, and odor.

*OVQ is scored as intuitional sensorial feeling that integrates quality parameters of appearance (yellowing, senescence browning, and wound browning/pinking), texture (crispness), and odor (smell; details are described in Supplementary Table 2).*

### Determination of Total Ascorbic Acid Content

Ascorbic acid was measured according to the method by Davey et al. (2003) with modifications. 300 mg fine ground sample from each pooled sample was extracted with 1.5 mL ice-cold 3.3% meta-phosphoric acid (MPA) and thawed on ice. The mixed solution was vortexed for 20 s and placed in ultrasonic bath at 0°C for 10 min in darkness. After 10 min centrifugation (25,000 rcf) at 4°C, 1 mL extract filtering through 0.45 μm filter was used for HPLC analysis of AsA. 100 μL filtered extraction was mixed with 50 μL of 5 mM dithiothreitol (DTT, in 400 mM Tris base) for converting DHA to AsA. After 15 min incubation in darkness and room temperature, 50 μl of 8.5% o-phosphoric acid was added into the mix to stop the reaction.

The concentration of AsA was analyzed using a HPLC consisting of a P580 pump (Dionex), a 340S UV-VIS detector (Dionex), and a MIDAS autosampler (Spark Holland) equipped with a ProntoSIL 120-3 C18 AQ, 250 × 3 mm column (Knauer) The column was eluted at a flow rate of 0.35 mL min^–1^ with 400 μL L^–1^ H_3_PO_4_ + 2.5 mL L^–1^ MeOH + 0.1 mM EDTA in H_2_O followed by a wash step with 30% acetonitrile in H_2_O. AsA was detected at 243 nm. The system was calibrated with standard AsA solution (prepared in 3% MPA/1 mM, stabilized with 2.5 mM DTT). The TAsA were calculated as the sum of the AsA directly measured and the AsA converted from DHA.

### Determination of Carbohydrate Content

Soluble sugars and starch were measured using a modified method according to van Geest et al. (2016). Carbohydrates were extracted from 15 mg freeze dried, fine ground sample with 5 mL 80% ethanol at 80°C in a shaking water bath for 20 min. After extraction, tubes were centrifuged (8,800 rcf) at 4°C and 1 mL supernatant was vacuum dried in a vacuum centrifuge (Savant SpeedVac SPD2010, Thermo Fisher Inc., Waltham, MA, United States) at 45°C and 5.1 mbar for 105 min. Then, 2 mL 0.01 M hydrochloric acid was added to re-dissolve the carbohydrates using an ultrasonic water bath (Branson 2200, Branson Ultrasonics, Danbury, CT, United States) at room temperature for 10 min. The solution was eluted over a HyperSep SCX cartridge, 100 mg/1 mL (Thermo Scientific, United States) to remove amino compounds and diluted 10 times with Milli-Q water for determination of glucose, fructose, and sucrose. The remaining pellets were stored in 80% ethanol at -20°C for starch analysis. The pellet was washed 3 times with 80% ethanol and then vacuum dried in a vacuum centrifuge at 45°C and 5.1 mbar for 25 min. 2 mL 1 g L^–1^ thermostable α-amylase (SERVA Electrophoresis GmbH, Heidelberg, Germany) was added to the dried pellet and incubated for 30 min at 90°C. After that, 1 mL solution of amyloglucosidase from *Aspergillus niger* (Sigma 10115, Sigma, St Louis, MO, United States; 0.5 mg mL^–1^ in 50 Mm citrate buffer, pH = 4.6) was added. After incubation at 60°C for 15 min, the solution was centrifuged (8,800 rcf) and diluted 20 times with Milli-Q water for quantification of the glucose. All soluble sugars were quantified using High Performance Anion Exchange Chromatography with Pulsed Amperometric Detection (HPAEC-PAD; Dionex ICS5000, Thermo Fisher Inc.), equipped with a Dionex CarboPac1 column (250 × 2 mm; Thermo Fisher), eluted with 100 mM NaOH at 0.25 mL min^–1^. Carbohydrates data before harvest were expressed on a fresh weight bases (g kg^–1^) similar to the TAsA level. Carbohydrate levels during postharvest were expressed on a dry weight bases (g kg^–1^) as fresh weight is subject to rapid changes when quality deteriorates in the later phase of storage which may obscure changes in absolute levels.

### Statistical Analysis

One-way analysis of variance (ANOVA) was used to test the effects of the light intensity on DM, FW, DM%, carbohydrates (sucrose, fructose, glucose, and starch) and TAsA for EXP 1 and EXP 2 when comparing different treatments at same time after treatment or time in shelf life. Normality of the variables was tested applying the Shapiro–Wilk test. Bartlett’s test was carried out to test homogeneity of variances. Fisher’s protected LSD was carried out for multiple comparison tests (*P* ≤ 0.01). Individual pooled samples consisted of 4 leaves from 4 different plants were considered as independent replicates, all measurements were based on four replicate samples. As there was only one compartment in the climate room per treatment, we may have underestimated the random variance. Therefore, the tests have been conducted at *P* = 99% instead of the commonly used *P* = 95%.

The Weibull distribution was fitted to the visual quality data according to Eq. 2 (Hertog, 2002; Ares et al., 2008) and were based on the average of 4 replicates in each treatments for OVQ scores (Figure 5) and on the individual replicates for individual visual quality traits (cut edge browning, senescence browning, yellowing/discoloring, odor/smell, and texture in Table 2).

(2) $$\begin{array}{c} F\left(t;b,c,d,e\right)=c+\left(d-c\right)\\ \left\{1-\exp\left\{-\exp\left[b\left(\log\left(t\right)-\log\left(e\right)\right)\right]\right\}\right\} \end{array}$$

Where *t* is the time after harvest; *b, c, d* and *e* are the model parameter estimates. Shelf life (*t*_shelf  life_) is calculated as the results when *F*(*t*;*b*,*c*,*d*,*e*) equals to 6 and are based on the individual replicates in each treatment. Statistical analysis was performed using the R software (R 3.4.3; R Project for Statistical Computing, Vienna, Austria).

## Results

### TAsA and Carbohydrate Levels at Harvest

**FIGURE 3:**
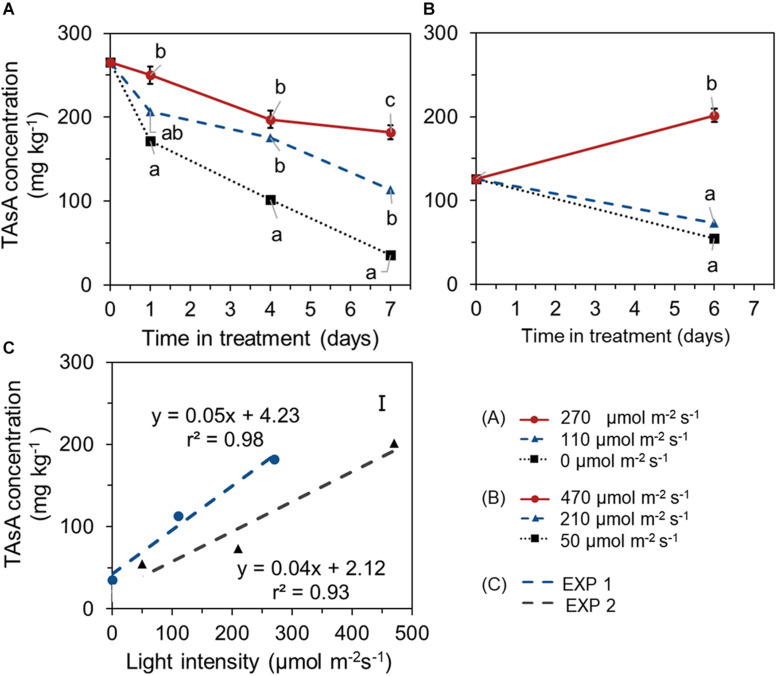
Time course of TAsA concentration (expressed on a fresh weight bases) of lettuce plants grown under different light intensities for the last 7 days in EXP 1 **(A)** or 6 days in EXP 2 **(B)** and the correlation between final TAsA concentration and light intensity **(C)**. EoP Light treatments started when plants were 5 weeks old. Data points represent means of 4 samples (*n* = 4), each consisting of leaves from 4 plants. Within each experiment, significant differences (at *P* < 0.01) are indicated by different letters when comparing different treatments at the same time point. Vertical bars represent standard errors of means [in panels **(A,B)** error bar only given in the highest line; in panel **(C)** error bar is given at right upper corner].

During the 1 week EoP light treatment, higher light intensity resulted in higher level of TAsA in lettuce leaves in both experiments. In EXP 1, TAsA concentration decreased over time of 7 days EoP light treatment at all light intensities compared to its start level at day 0. However, the decrease of TAsA concentration was stronger the lower the light intensity (Figure 3A). In EXP 2, plants from treatment with 210 μmol m^–2^ s^–1^ intensity (same light intensity as during growth) showed a decrease in TAsA level over treatment time. Only at the highest light intensity (470 μmol s^–1^m^–2^ for 6 days), TAsA was significantly increased at the end of the EoP light treatment (Figure 3B). A linear relation was found between the light intensity (applied as EoP lighting) and the level of TAsA at the end of the light treatment (Figure 3C).

**FIGURE 4:**
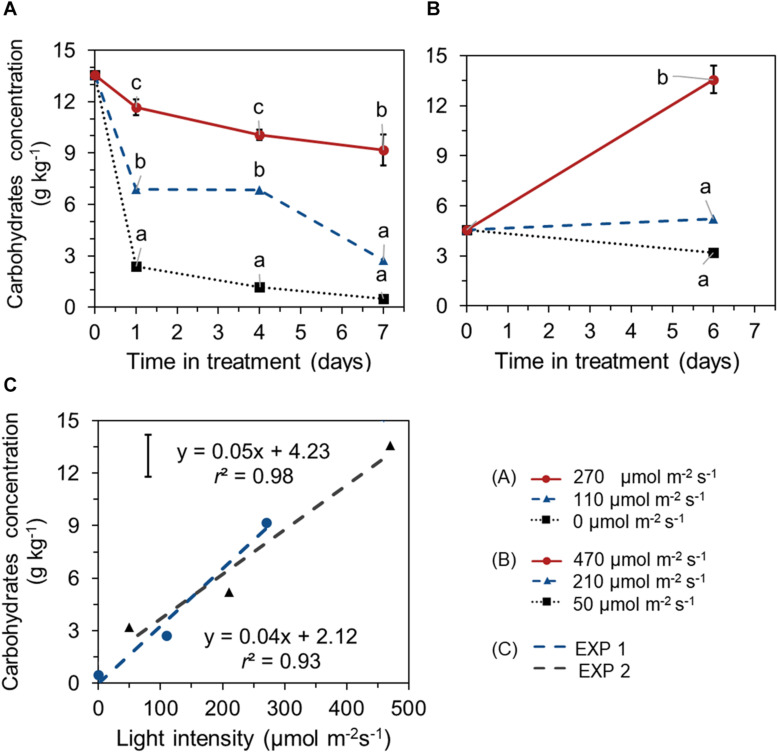
Time course of total carbohydrate concentration (sum of glucose, sucrose, fructose, and starch, expressed on a fresh weight bases) of lettuce plants grown under different light intensities for the last 7 days in EXP 1 **(A)** or 6 days EXP 2 **(B)** and the correlation between final carbohydrate level and light intensity **(C)**. Light treatments started when plants were 5 weeks old. Data points represent means of 4 samples (*n* = 4), each consisting of leaves from 4 plants. Within each experiment, significant differences (at *P* < 0.01) are indicated by different letters comparing different treatments at the same time point. Vertical bars represent standard errors of means [in panels **(A,B)** error bar only given in the highest line; in panel **(C)** error bar is given at left upper corner].

Different EoP light intensities also affected the level of total carbohydrates (glucose + fructose + sucrose + starch). In EXP 1, carbohydrates concentration in leaves showed a decreasing trend in all treatments and reached significant lower values at day 7 compared to its start level at day 0. Carbohydrates reduction was less under higher light intensity (270 μmol s^–1^ m^–2^). A steep decline in carbohydrates was observed in darkness (Figure 4A). In EXP 2, for lettuce that was grown under 210 μmol s^–1^ m^–2^ light during both the initial stage and EoP lighting stage, no significant changes in carbohydrates level were shown with crop development during the EoP light treatment. At the highest light intensity (470 μmol s^–1^ m^–2^), the carbohydrate level substantially increased (Figure 4B). Carbohydrate levels at the end of EoP light treatments were linearly correlated with applied light intensities in both experiments (Figure 4C). Glucose, sucrose, fructose, and starch levels showed similar responses to light intensity as the total carbohydrates level (Supplementary Figure 1).

In both experiments, increased light intensity during the last week of cultivation significantly increased the dry weight and dry matter percentage of lettuce leaves, where fresh weight only showed a slight increase in EXP 2 (Supplementary Figure 2).

### TAsA and Carbohydrate Levels During Shelf Life in Darkness

**FIGURE 5:**
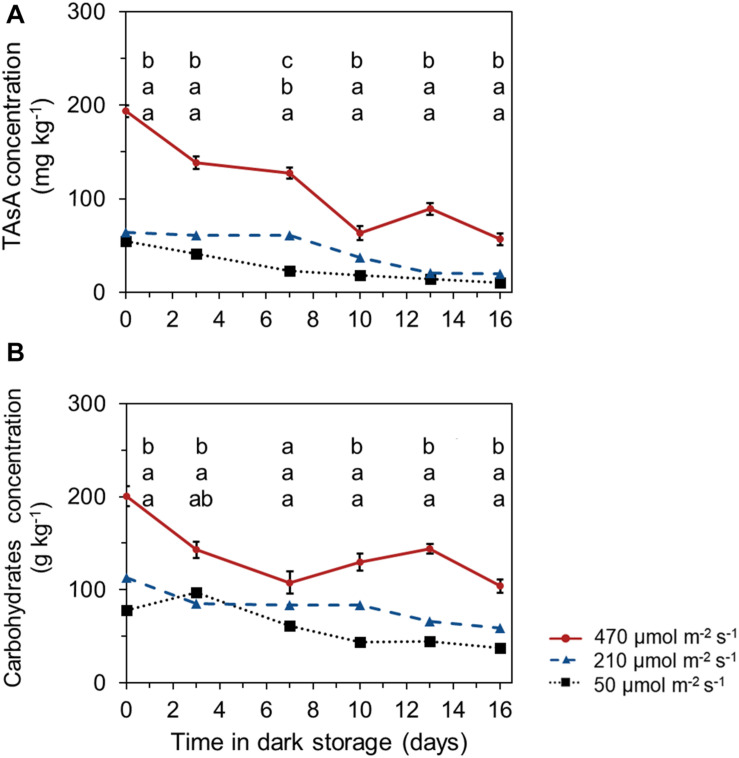
Time course of TAsA **(A)** and total carbohydrate (glucose + fructose + sucrose + starch) levels **(B)** during shelf life at 10°C in darkness. The TAsA concentration was expressed on fresh weight bases; carbohydrate concentration was expressed on a dry weight bases. Samples were derived from plants that received different pre-harvest lighting intensities (50, 210 and 470 μmol m^– 2^ s^– 1^) for 6 days (EXP 2). Data points represent means of 4 samples (*n* = 4), each consisting of leaves from 4 plants. Within each experiment, significant differences (at *P* < 0.01) are indicated by different letters comparing different treatments at the same time point. Vertical bars represent standard errors of means (only given in the highest line).

Both TAsA and total carbohydrate levels declined during the shelf life in darkness. The leaves harvested from plants that received the highest pre-harvest light intensities, maintained a significant higher TAsA and total carbohydrate level during the whole shelf life period until the end of storage (Figures 5A,B). Postharvest levels of glucose, sucrose, fructose, and starch showed similar trends as the total carbohydrate levels (Supplementary Figure 3).

### Overall Visual Quality and Shelf Life

**FIGURE 6:**
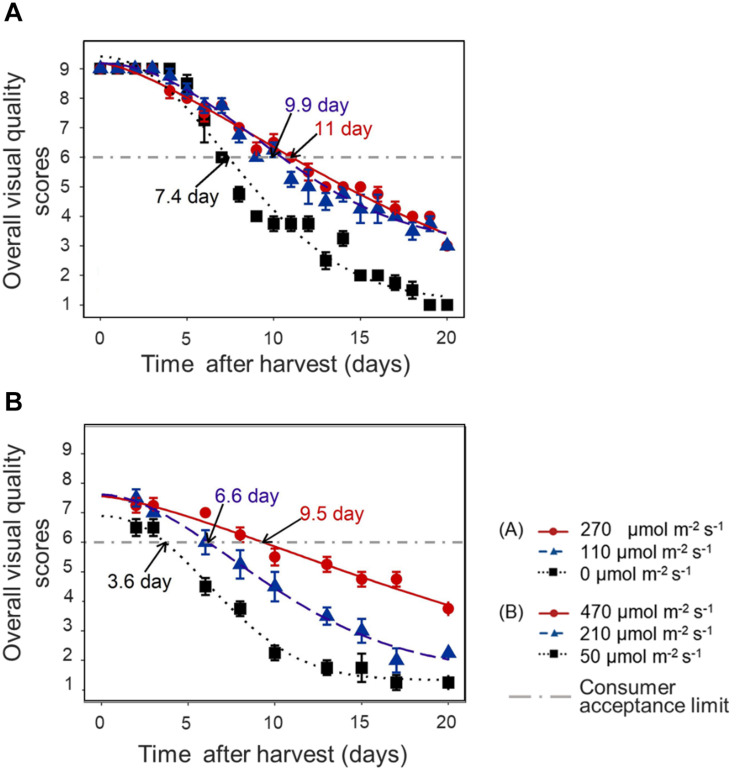
Time course of lettuce OVQ changes during shelf life at 10°C in darkness in EXP 1 **(A)** and EXP 2 **(B)**. End of Production (EoP) light treatment was applied to 5 weeks old plants and lasted 7 (EXP 1) or 6 days (EXP 2). Data points represent means of 4 samples (*n* = 4), each sample consisting of 4 leaves from 4 plants. The curves show the fitted model according to Eq. 2 and are based on the average of 4 replicates in each treatment. The horizontal dash-dot line indicates the defined consumer acceptance threshold (OVQ score = 6). Shelf life was calculated from the intersection of fitted Weibull curves and consumer acceptance threshold, numbers indicated by arrows are average of 4 estimated shelf life figures. Error bars indicates standard error of the mean measured value.

In both experiments, the decline of OVQ was suppressed by applying increased light intensity in the last week before harvest. This resulted in a significantly extended shelf life. Pre-harvest light intensity of 110–270 μmol m^–2^ s^–1^ increased the shelf life by 3–4 days compared to darkness in EXP 1 (Figure 6A) and increasing light intensity to 470 μmol m^–2^ s^–1^ increased the shelf life by 6 days compared to low light condition (50 μmol m^–2^ s^–1^) and by 3 days compared to moderate light condition (210 μmol m^–2^ s^–1^) in EXP 2 (Figure 6B).

In EXP 2, the dynamics of individual quality aspects was further analyzed. Table 4 shows the time that respective quality aspects reach the consumer acceptance limit. Shelf life (based on OVQ) appeared to be primarily determined by leaf yellowing and senescence browning.

**TABLE 4:** Effect of pre-harvest light intensity on time until consumer acceptance limit was reached according to scores of different quality traits in EXP 2.

Light intensity (μ mol m^–2^ s^–1^)	Time to consumer acceptance limit (days)					
	OVQ	Cut edge browning	Senescence browning	Yellowing/Discoloring	Odor/Smell	Texture
50	3.6 ± 0.50	6.4 ± 0.59	4.2 ± 0.58	3.0 ± 1.02	4.9 ± 0.71	4.4 ± 0.84
210	6.6 ± 0.72	7.0 ± 0.77	7.1 ± 0.44	5.8 ± 0.95	6.0 ± 1.26	7.7 ± 0.37
470	9.5 ± 0.52	11.2 ± 2.48	8.5 ± 0.98	8.1 ± 0.74	10.8 ± 1.35	12.4 ± 0.54

*Values represent the average of 4 estimated values (n = 4) according to fitted Weibull curve; each value is based on a pooled sample consisting of leaves from 4 plants. Values are mean ± standard error of the mean measured value.*

### Pre-harvest Light Intensity and Nutritional Quality at Harvest Is Positively Corelated With Shelf Life

**FIGURE 7:**
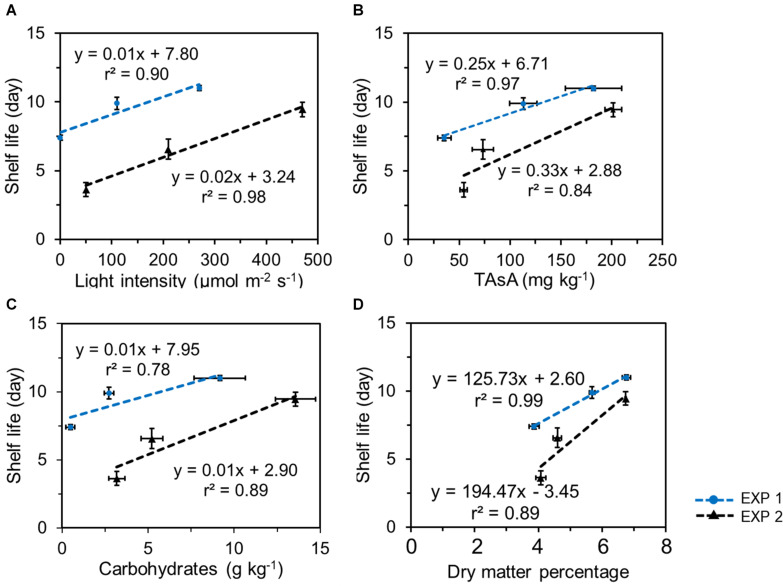
Correlations between lettuce shelf life and light intensity applied in pre-harvest treatment **(A)**, the total ascorbic acid level (**B**, expressed on a fresh weight bases), the total carbohydrates (glucose + fructose + sucrose + starch) levels (**C**, expressed on a fresh weight bases), and dry matter percentage **(D)** in lettuce at harvest in 2 experiments. Plotted values represent the average value of 4 samples (*n* = 4) each consisting of leaves derived from 4 different plants. Vertical bars indicate standard errors of the mean estimated shelf life; horizontal error bars indicate standard errors of the mean carbohydrate content and of dry matter percentage.

In both EXP 1 and EXP 2, the shelf life was positively correlated with the EoP light level and with the nutritional quality level at harvest, both with respect to the levels of TAsA and total carbohydrates (Figure 7). Similar correlations were found between shelf life and the levels of individual soluble sugars and total soluble sugar at harvest (Supplementary Figure 4; glucose 2/J, fructose 3/J, sucrose 4/J, and total soluble sugar 5/J). In addition, a good correlation was observed between the shelf life and the dry matter percentage at harvest (Figure 7D), the latter being directly related to the improved levels of carbohydrates.

## Discussion

Most of the previous research on the effects of light intensity on end product quality has been done using different light levels applied during the entire cultivation period. Here we focussed on applying different light levels only at the days before harvest. This has the advantage that the light treatments have limited influence on crop growth, yield and morphology. In addition, EoP lighting limits the energy input required for higher light intensities compared to prolonged lighting.

We showed that short-term (6 or 7 days) high light intensity applied in the days before harvest significantly increases the nutritional quality in lettuce. This is reflected in higher levels of carbohydrates and TAsA. The improved nutritional quality is maintained during the postharvest phase and positively affects postharvest performance and the shelf life.

### High End of Production Light Intensity Improves Nutritional Status at Harvest

The effects of high light during growth on plant nutritional quality at harvest have been studied before (Zhou et al., 2009; Locato et al., 2013). For instance, lettuce grown under light intensities above 200 μmol m^–2^ s^–1^ (red and blue LEDs) showed increased antioxidants capacity and higher phenolic and flavonoids levels at harvest compared to plants grown under light intensities of 100 and 150 μmol m^–2^ s^–1^ (Pennisi et al., 2020).

High light intensities applied as EoP lighting was shown before to improve the nutritional status at harvest. Carbohydrates and chlorophyll content in harvested lettuce were increased when increasing the light intensity from 400 to 700 μmol m^–2^ s^–1^ in the 4 days before harvest (light source not mentioned; Pérez-López et al., 2015). The level of soluble sugars and starch in lettuce increased 1.5 times at harvest when additional supplemental lighting (1,000–1,200 μmol m^–2^ s^–1^ from HPS lamps) is applied in greenhouse 10 days before harvest (Zhou et al., 2009). Samuolienë et al. (2011) showed that application of supplemental red LEDs (300 μmol m^–2^ s^–1^) during last 3 days before harvest observed increasing TAsA in lettuce at harvest.

In our results, we found a positive correlation between carbohydrates level and TAsA level. This was also found in previous research about light regulation of TAsA (Nishikawa et al., 2005; Zhou et al., 2009; Schmitz et al., 2014; Pérez-López et al., 2015), however, the causal connection between carbohydrates levels and the increases in TAsA has not yet been proven (Yabuta et al., 2007). Although glucose is the starting point for AsA biosynthesis, more sugars does not automatically lead to more AsA (Ntagkas et al., 2019). The activities and expression of many enzymes and genes involved in TAsA biosynthesis and recycling pathway are induced under high light intensity in both leaves and in fruits (Bartoli et al., 2006; Dowdle et al., 2007; Yabuta et al., 2007). The light regulation of these enzymes and genes is suggested to be mediated by photosynthesis (through its effect on the plastoquinone state) and by respiration (Bartoli et al., 2006; Ntagkas et al., 2018). Both the increase in carbohydrate and AsA during high intensity EoP lighting may therefore result from the increased photosynthetic activity.

### High EoP Light Improves the Postharvest Performance and Shelf Life

Our results showed that in all treatments, carbohydrates and TAsA levels decreased during dark storage. However, plants which had received high light intensity before harvest, showed higher starting levels of these compounds and maintained higher levels throughout the entire storage period (Figure 6). This resulted in an improved visual quality and a longer shelf life.

The rapid deterioration that occurs in leafy material during dark storage is due to a phenomenon called “dark induced senescence,” primarily induced by a developing shortage of carbohydrates (Van Doorn, 2004a). Senescence itself is a form of programmed cell death in which the cells degrade their contents to sustain energy production before they die (Van Doorn and Woltering, 2004). The later phase of senescence is often accompanied by an increased production of ROS and associated loss of membrane integrity (Van Doorn and Woltering, 2005). These processes lead to a sequential loss of organelles, chlorophyll, proteins and other compounds and finally death of the cells. This is reflected in loss of sensorial quality (e.g., loss greenness, loss of shininess and crispness, tissue collapse, and browning; Thimann et al., 1977; Büchert et al., 2011). A higher nutritional status at the start may postpone such deteriorative processes during storage.

The reduction of TAsA after harvest is caused by both increased consumption and decreased biosynthesis (Smirnoff and Wheeler, 2000). On the one hand, TAsA is used to scavenge ROS, which are usually generated under postharvest conditions (e.g., sugar starvation, wounding and leaf senescence; Smirnoff, 2000; Nishikawa et al., 2003; Couée et al., 2006; Baxter et al., 2014). In this way TAsA can protect against oxidative stress. On the other hand, substrates for TAsA biosynthesis may be limited during dark storage, possibly due to absence of photosynthesis, chloroplast disintegration, and interruption of carbohydrates allocation from source leaves (Nishikawa et al., 2003). Limited carbohydrate availability may suppress AsA biosynthesis and recycling through suppression of the genes involved in AsA biosynthesis and recycling (Millar, 2003; Nishikawa et al., 2005).

We found positive correlations between the shelf life and the contents of carbohydrates and TAsA at harvest (Figure 7), indicating that the higher nutritional quality indeed postponed the deteriorative processes during postharvest storage. Previous studies showed that higher sugar levels in plants suppressed yellowing in broccoli florets (Coupe et al., 2003; Nishikawa et al., 2005), reduced petal senescence and increased keeping quality in cut flowers (Fjeld et al., 1994; Van Doorn, 2004b; Fanourakis et al., 2013; Pattaravayo et al., 2013). Additionally, visual quality deterioration in lettuce was greatly delayed when the product was stored under low light intensities (5 to 30 μmol m^–2^ s^–1^ provided by fluorescent tubes or narrow band red, blue, red + blue, or green LEDs) compared to dark storage (Zhan et al., 2013; Woltering et al., 2015, 2016; Woltering and Witkowska, 2016), and this was found to be associated with increased levels of sugars and TAsA under postharvest lighting.

In our research, cut edge browning and senescence browning was suppressed in leaves that contained higher levels of TAsA as a result of high light intensity applied in EoP light treatment (Supplementary Figure 4. 1/L and 1/M). The anti-browning effects of TAsA have been shown in previous research on fruit and leaves (Soliva-Fortuny and Martín-Belloso, 2003; Degl’Innocenti et al., 2005). TAsA inhibits enzymatic browning (pink/brown coloration mainly on cut surface) by competing with PPO and reducing colored quinones products to colorless diphenols (Couture et al., 1993; Degl’Innocenti et al., 2005, 2007).

The improved postharvest performance and prolonged shelf life of the leaves from high intensity EoP lighting is likely a direct result of the higher levels of carbohydrates and TAsA. Both compounds postpone deteriorative processes connected to senescence and AsA, in addition, may play a role in limiting tissue browning.

### Future Perspective

End of Production light treatments seem a feasible way to improve plant nutritional and health related properties and can be employed to improve postharvest performance and shelf life of leafy vegetables. Here we studied the effect of EoP light intensity (red – white LEDs) during 1 week and a photoperiod of 16–18 h. Other light factors, such as photoperiod and spectrum and the optimal EoP light duration need to be studied to further elucidate the underlying mechanisms of light effects on crop nutritional status and postharvest performance.

## Conclusion

Increased light intensity at the EoP phase increased the levels of carbohydrates, TAsA and the percentage of dry matter at harvest, indicating an improved nutritional quality of the lettuce. The response to EoP lighting was independent on the cultivation history in either a greenhouse or vertical farm. The improved nutritional status of the lettuce was maintained during the subsequent postharvest storage. The higher levels of carbohydrates and TAsA postpone deteriorative processes connected to senescence, in addition, TAsA may play a role in limiting tissue browning. The improved nutritional status at harvest resulted in a better postharvest performance and extended the shelf life.

## Data Availability Statement

The raw data supporting the conclusions of this article will be made available by the authors, without undue reservation.

## Author Contributions

QM, EW, CN, and LM conceptualized the research plan. QM, EW, and LM designed the experiments and QM carried out the experiments, analyzed the data, and wrote the manuscript. EW and LM provided critical feedback on the manuscript. CN provided critical comments to the overall structure of the manuscript. All authors reviewed and approved the final manuscript.

## Conflict of Interest

The authors declare that the research was conducted in the absence of any commercial or financial relationships that could be construed as a potential conflict of interest.
