# Supplementary material for: High Light Intensity Applied Shortly Before Harvest Improves Lettuce Nutritional Quality and Extends the Shelf Life
Source: Front Plant Sci. 2021 Jan 28;12:615355. doi: 10.3389/fpls.2021.615355 (PMC7876091; doi:10.3389/fpls.2021.615355)
Supplement: Supplementary file 1 [file Data_Sheet_1.docx]

Supplementary Material

# Supplementary Tables

| **Supplementary Table 1.** Measured LED light intensity (µmol s^-1^m^-2^) in 100 nm bands wavelength in EXP 1 and EXP 2. | | | | | | | | |
| --- | --- | --- | --- | --- | --- | --- | --- | --- |
|  | EXP 1 | | | EXP 2 | | | | |
|  | EoP treatments | | | Growth phase | | EoP treatments | | |
|  | High | Medium | Dark | +5% FR | without FR | High | Medium | Low |
| 400-500 nm | 44.4 ± 1.33 | 17.97 ± 14.49 | 0 | 36.50 ± 1.20 | 36.50 ± 1.20 | 80.91 ± 3.27 | 36.50 ± 1.12 | 7.36 ± 0.26 |
| 501-600 nm | 68.71 ± 1.85 | 27.75 ± 3.15 | 0 | 54.56 ± 1.67 | 54.56 ± 1.67 | 119.51 ± 4.37 | 54.56 ± 1.56 | 13.14 ± 0.39 |
| 601-700 nm | 152.42 ± 4.13 | 63.50 ± 7.05 | 0 | 120.06 ± 3.61 | 120.06 ± 3.61 | 268.48 ± 9.95 | 120.06 ± 3.36 | 28.81 ± 0.84 |
| 701-800 nm | 2.72 ± 0.09 | 0.87 ± 0.15 | 0 | 13.6 ± 0.31 | 2.05 ± 0.09 | 4.94 ± 0.21 | 2.05 ± 0.08 | 0.36 ± 0.03 |
| PFD ^a^ | 268.26 ± 7.29 | 110.08 ± 12.47 | 0 | 224.72 ± 6.80 | 213.17 ± 6.10 | 473.84 ± 17.78 | 213.17 ± 6.10 | 49.67 ± 1.48 |
| PPFD ^b^ | 265.54 ± 7.21 | 109.21 ± 12.32 | 0 | 211.12 ± 6.02 | 211.12 ± 6.02 | 468.9 ± 17.58 | 211.12 ± 6.02 | 49.31 ± 1.46 |
| a. PFD, photon flux density (400 - 800 nm). | | | | | | | | |
| b. PPFD, photosynthetically photon flux density (400 - 700 nm). | | | | | | | | |

| **Supplementary Table 2.** The description visual quality scoring scale in appearance (yellowing, senescence browning and cut edge browning), texture and odour /smell for lettuce leaf (cv. Expertise). | | | | | |
| --- | --- | --- | --- | --- | --- |
|  | ***Appearance*** | | | ***Texture  (crispness)*** | ***Odour/Smell*** |
|  | ***Yellowing / Discolouring*** | ***Senescence browning*** | ***Cut edge browning*** |  |  |
| ***9- Excellent*** | Exceptionally appealing, bright, and typical natural green colour for the cultivar. | Bright and typical natural green at the leaf blade, fresh and white at the stem. | Fresh and bright at cutting edge, no brown was shown. | Exceptional good at typical characteristic texture: firm and crispy. | Exceptional delicate, distinct odour, e.g. fresh\sweet grass smell. |
| ***8- Very good*** | Bright and typical natural green colour, single slightly yellow/discoloured blade allowed. | Typical natural green on the blade, fresh and white at the stem. | Less bright at cutting edge, slightly pink/brown shown on the cutting edge. | Very good at firm and crispy. | Full, strong odour, e.g. fresh grass smell. |
| ***7- Good*** | Bright and typical natural green colour, a few slightly yellow/discoloured blades allowed. | Natural green at the most leaves, single slightly brown at the leaf blade/stem allowed. | Obvious slightly brown was shown on the cutting edge. | Good in firm and crispy. | Reduced in typical grass odour, no unpleasant odour. |
| ***6- Satisfactory*** | Slight reduction of brightness, slightly yellow/discoloured specimen allowed. | Natural green on the most leaves, slightly brown at the leaf blade and stem, not yet spread. | Widespread moderate brown shown on the cutting edge. | Normal at firm and crispy. Slightly soft leaf allowed. | No unpleasant odour. |
| ***5- Mediocre*** | Reduction in typical green colour, few more yellow on leaf blade, unbalanced colour. | Brown spread at leaf blade or midribs. | Widespread darker brown was shown, cutting edge can be wet. | Impairment of texture in firm a crispy, still acceptable. | Slightly unpleasant odour emerged, e.g. sour\fermented smell. |
| ***4- Borderline*** | Obvious yellowing and discolouration on the leaf blade, not yet unpleasant. | Brown spread at both leaf blade and midribs, brown tissue getting soft. | Slightly water leakage appears at the brown cutting edge. | Clearly reduced firmness and crispiness, soft at leaf. | Unpleasant odour emerged, e.g. sour\fermented smell. |
| ***3- Poor*** | Strong discolouration and obvious yellow on the leaf blade. | Obvious brown at both leaf blade and midribs, brown tissue getting soft and wet. | Water leakage appears at the brown cutting edge. | Leaf getting welted and soft. | Obvious unpleasant odour, e.g. sour, fermented, or spoiled smell. |
| ***2- Bad*** | Complete yellowed\white\browned on the leaf blade, original colour no longer perceptible. | Strong brown at both leaf blade and midribs, most of the tissue discoloured and perished. | The wound starts perishing, accompanied with softening and liquid leakage. | Unpleasantly in texture, soft and water leakage may appear. | Strong unpleasant odour, e.g. sour, fermented, or spoiled smell. |
| ***1- Very bad*** | Complete discoloured on the leaf blade towards repulsive colours. | Complete discoloured and perished. | The wound completely brown and leaking liquid. | Repulsive in texture. | Repulsive, strange odour, e.g. spoiled, alcohol and fermented smell. |

# Supplementary Figures

| 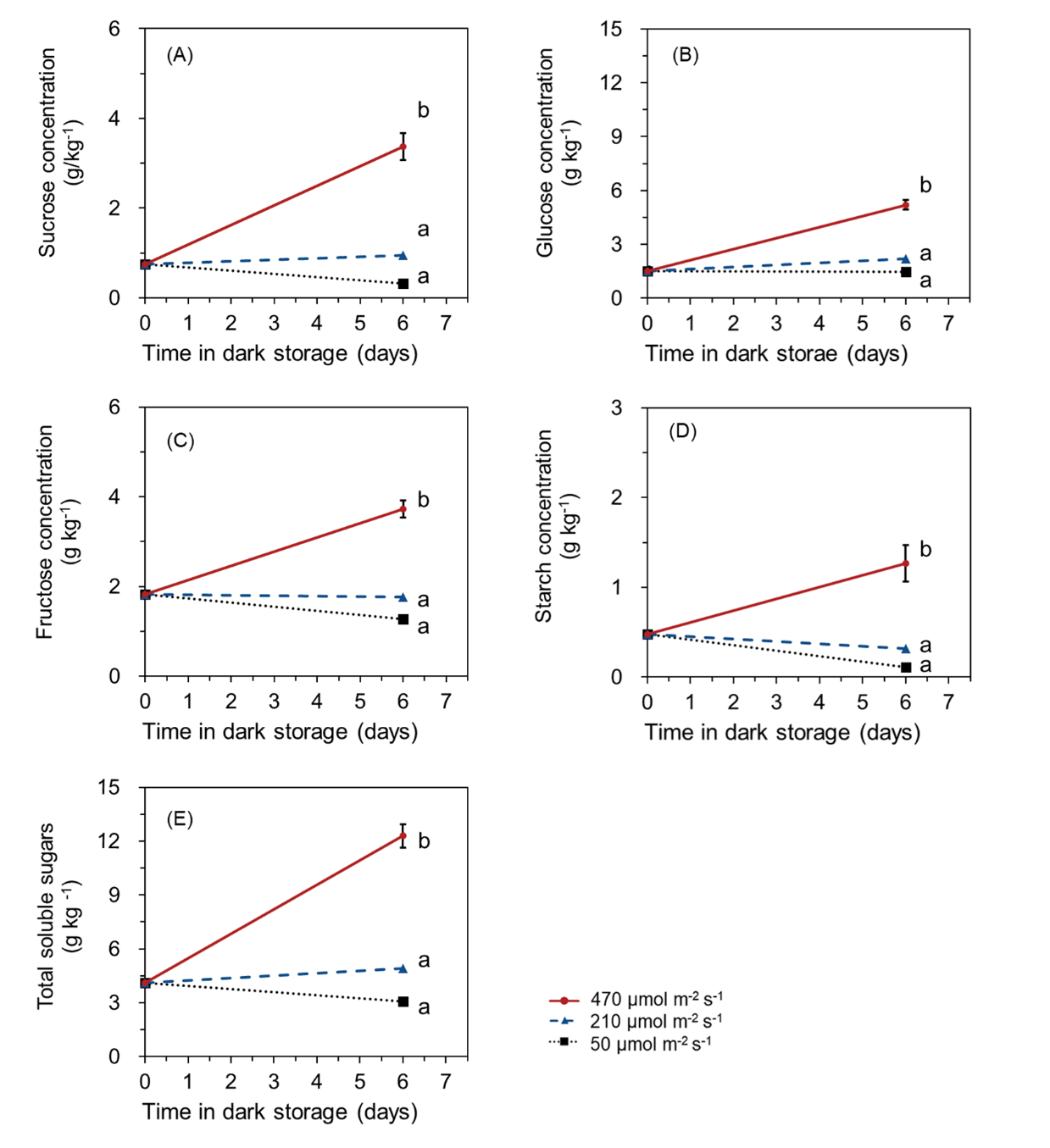 |
| --- |
| **Supplementary Figure 1.** Time course of sucrose (A), glucose (B), fructose (C), starch (D) and total soluble sugars (sucrose + glucose + fructose) levels as affected by End of Production (EoP) light treatment in EXP 2. The concentrations of sugars were expressed on a fresh weight bases. Samples were derived from plants that received different EoP lighting intensities (50, 210 and 470 µmol m^-2^s^-1^) for six days. Data points represent means of 4 samples (n = 4), each sample consisting of leaves from 4 plants. Within each experiment, significant differences (at P < 0.01) are indicated by different letters comparing different treatments at the same time point. Vertical bars represent standard errors of means (only given in the highest line). |

| 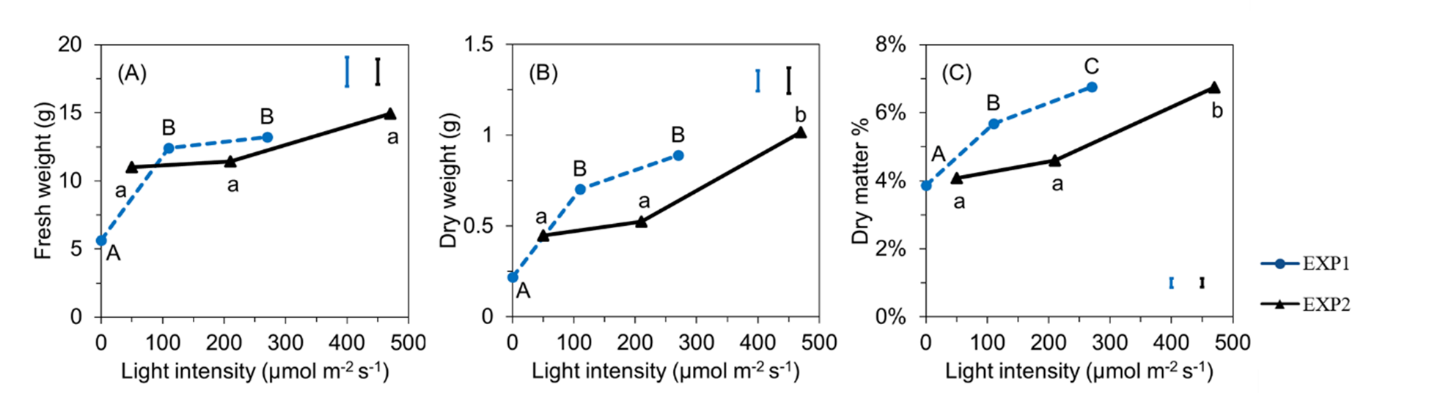 |
| --- |
| **Supplementary Figure 2.** Effect of light intensities applied in short-term pre-harvest light treatments on lettuce fresh weight (A), dry weight (B) and dry matter % (C) at harvest. Pre-harvest treatment was applied to 5 weeks old plants and lasted 7 (EXP 1) or 6 days (EXP 2). The initial (before the End of Production light treatments) levels of fresh weight, dry weight and dry matter percentage were 12.15 ± 0.56 g, 0.83 ± 0.04 g and 6.8 % ± 0.06 % in EXP 1 and 11.44 ±0.38 g, 0.52 ± 0.02 g, 4.6 % ± 0.2 % in EXP 2. Data points represent means of 4 samples (n=4), each samples consisting leaves from 4 plants. Within each experiment, significant differences (at *P* < 0.01) between each light intensity levels are indicated by letters in uppercase (EXP 1) and lowercase (EXP 2). Vertical bars represent standard errors of means. |

| 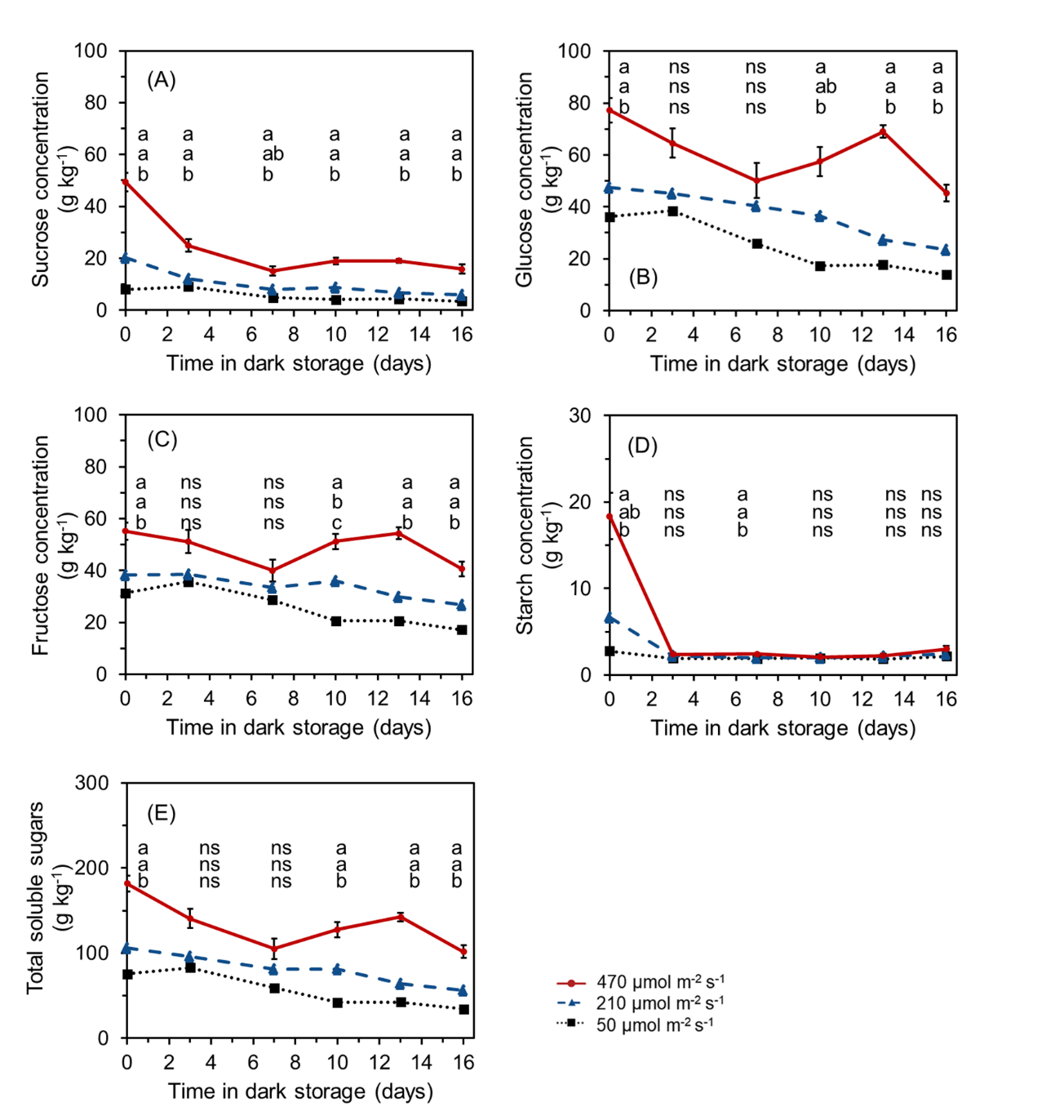 |
| --- |
| **Supplementary Figure 3.** Time course of sucrose (A), glucose (B), fructose (C), starch (D) and total soluble sugars (sucrose + glucose + fructose) levels during shelf life at 10 ℃ in darkness. The concentrations of sugars were expressed on a dry weight bases. Samples were derived from plants that received different End of Production light intensities (50, 210 and 470 µmol m^-2^s^-1^) for six days (EXP 2). Data points represent means of 4 samples (n = 4), each sample consisting of leaves from 4 plants. Within each experiment, significant differences (at P < 0.01) are indicated by different letters comparing different treatments at the same time point, non-significant differences are indicated as ns. Vertical bars represent standard errors of means (only given in the highest line). |

| 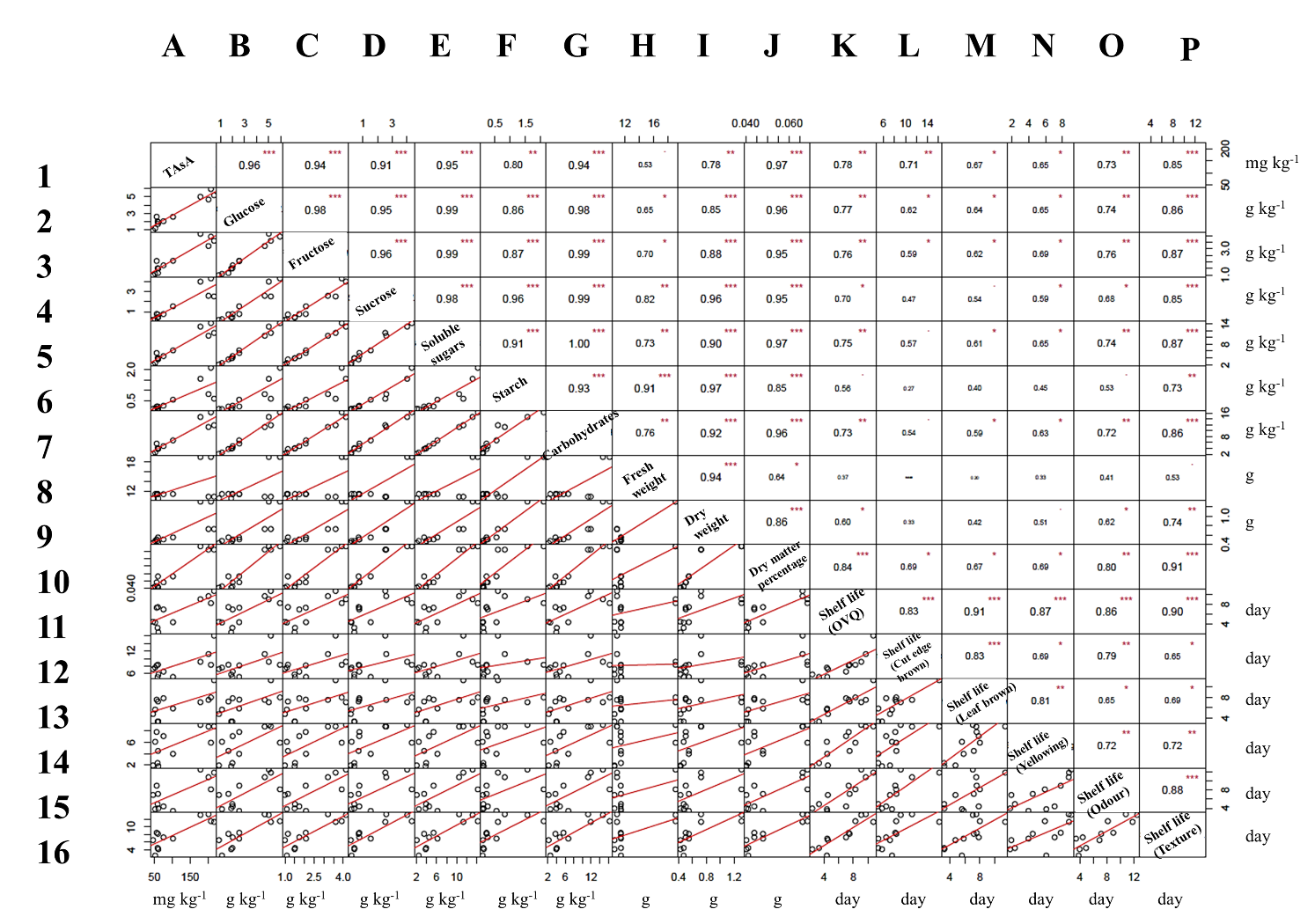 |
| --- |
| **Supplementary Figure 4.** Pearson's correlation matrix of nutritional quality level at harvest, crop growth properties at harvest and estimated shelf life in EXP2. From column A to Q, nutritional quality including TAsA, glucose, sucrose, fructose, starch, total soluble sugars and carbohydrates; crop growth properties including fresh weigh, dry weight and dry matter percentage; estimated shelf life is calculated base on overall visual quality (OVQ) score, cut edge browning score, senescence browning score, yellowing/discolouring score, odour/smell score and texture score. Scatter plots with linear trend line are shown in the lower panel with linear trend line and correlation coefficients are shown in the upper panel. Correlation significance are indicated with *** (p < 0.001), **(p < 0.01), * (p < 0.05), ∙ (p < 0.1). Data points represent individual samples (n = 16). |
